# The Efficiency of Sulphuric Acid in Diarrhœa

**Published:** 1854-04

**Authors:** Goodeve Bowra


THE EFFICIENCY OF SULPHURIC ACID IN DIARRHŒA.—By GOODEVE BOWRA, ESQ., M. R. C. S. E.—As much has been said and written lately on the treatment of diarrhœa, I beg to offer my testimony on the happy effects of sulphuric acid on persons of all ages, with the full conviction that it is the quickest and most palatable, consequently the best remedy for that disease.

I was requested two years ago to send medicine to a young lady subject to diarrhœa, who had taken a passage to China in a ship not carrying a surgeon. Thinking there must be some mistake, I went on board the vessel and saw the chief officer, who thought himself quite competent to treat (from books and a medicine chest) any disorder likely to arise on the voyage. On asking this gentleman what he would do in cases of diarrhœa, his off-hand reply was, "Never care for that; always carry plenty of sulphuric acid on board, and I have never known it fail."

On the same evening I was called to a lady, seventy-five years of age, very subject to these attacks, which always lasted a week or more, and as it was of great consequence she should return into the country in two days, I made up my mind to try the sulphuric acid, believing I should not be more successful with the old chalk mixture than her medical friend at home.

The following morning I found her up, and so much better, that she had only taken two doses; I gave her a third, which completely cured her. Since then I have used nothing else (save a mustard poultice) in all cases, either with or without pain, both in private practice, and an institution to which I was attached, and I confidently state that during the whole period I have used it, I have not met with one unsuccessful case. The only difficulty is, to persuade some people that acids would not increase their disorder, particularly those accustomed to the old chalk and aromatic confection treatment.

I now constantly recommend patients subject to diarrhœa, or who are nervous about cholera, to keep a bottle of the sulphuric acid mixture in the house, and on the first symptom of their complaint instantly to take a dose, which is generally sufficient to effect a cure. Many of these patients (I fear, jokingly) tell me they shall expect double charges, as two or three draughts of the acid mixture have more effect than the same number of bottles of the chalk mixture; and I may add, I feel so satisfied with the success of a two years' trial, that I have no hesitation in asserting it as my full belief, that deaths from cholera and diarrhœa would be very materially diminished if the authorities would appoint an agent in all poor neighborhoods, to give a dose of the sulphuric acid mixture to every necessitous applicant suffering from bowel complaint. They would have plenty of persons desirous of availing themselves of this remedy, if I may judge from the gallons I gave away last summer.—*Lancet.*

